# The treatment-related experiences of parents, children and young people with regular prescribed medication

**DOI:** 10.1007/s11096-018-0756-z

**Published:** 2018-11-26

**Authors:** Jeff Aston, Keith A. Wilson, David R. P. Terry

**Affiliations:** 1 Aston Pharmacy School, Aston University, Birmingham, UK; 2 Pharmacy Department, Birmingham Women’s and Children’s NHS Foundation Trust, Birmingham, UK

**Keywords:** Drug therapy, Medication therapy management, Paediatrics, Qualitative research, Self management, United Kingdom

## Abstract

*Background* Taking regular medication has been shown to have an impact on the daily lives of patients and their families. *Objective* To explore the medication-related experiences of patients and their families when a child or young person is prescribed regular medication. *Setting* A specialist U.K. paediatric hospital. *Method* Semi-structured face-to-face interviews of 24 parents/carers, children or young people, who had been taking two or more medications for 6 weeks or longer. The themes explored included the medication regimen, formulation, supplies, social aspects and adverse effects. The data was analysed using NVIVO version 11. *Main outcome measure* The experiences of patients, and their parents/carers, when a child/young person takes regular medication. *Results* Participants described a range of experiences associated with taking regular medication. Medication-related challenges were experienced around the timing of administration which was managed over 24 h rather than waking hours. Updating medication doses for administration at school was often delayed. Unintended nonadherence was cited as the biggest challenge with a range of strategies employed to manage this. The internet was commonly used as a source of additional information accessed for reassurance and adverse effects but there were varying experiences of using patient forums/help groups. Other challenges included the adequacy of information, travelling with medication, formulation issues, arranging supplies and adverse effects. *Conclusion* Patients and parents experience many challenges with children’s medication. Individualised treatment options should be considered. Further research is required to determine how these experiences may be managed including the role of paediatric medication review.

## Impacts on practice

- Patients and their parents experience a range a medication-related challenges when a child takes regular medication.
- Pharmacists and other healthcare professionals, by being aware of these challenges, have the opportunity to further optimise medication use in this population.

## Introduction

Efforts to assist patients with adherence might improve the benefits of prescribed medication [1]. In children, adherence may be influenced by parents’/carers’ beliefs about the condition, regimen, child resistance, daily life and health professional influence [2].

Taking medication has been shown to place a burden on patients’ daily lives including the routine of taking medication, monitoring and travelling [3, 4]. The formulation, quantity, packaging, brand, adverse effects and negotiating the healthcare system add to this burden [3]. The stigma from family and friends associated with taking medication may add a psychological burden and influence patients’ beliefs about medication [3].

The experiences of children, and families, taking medication have been described for asthma [5, 6], diabetes [6], cystic fibrosis [7, 8], attention deficit hyperactivity disorder [9], inflammatory bowel disease [10], diabetes [11] and post-transplant patients [12, 13]. Challenges were described around medication use in school, taking in front of peers, social activities, regimen rigidity, reliance on family and adherence. The desire to achieve normality in adolescents can lead to patient-initiated changes to their medication [14].

Treatment burden can lead to poor adherence, waste and poor outcomes [15]. Minimally disruptive medication tailored to the realities of patients’ daily lives could greatly improve quality of life [15]. For children and young people, understanding how medication taking affects daily life may help identify opportunities for optimising use.

### Aim of the study

To explore the treatment-related experiences when children and young people take regular prescribed medication.

### Ethics approval

Approved by the West of Scotland Research Ethics Committee 16/3/17, reference 17/WS/0038.

## Method

This study was undertaken at Birmingham Children’s Hospital -a UK paediatric hospital.

Purposive sampling by ward pharmacists of in-patients aged up to 18 years who had been taking two or more prescribed medications concurrently at home, prior to admission, for 6 weeks or longer. Each participant was provided with an information sheet. Participants who wished to join the study were identified to JA who took consent. Consent was taken from the patient’s parent/carer who acted as the study participant if the child was under 16 years old or the patient if aged 16 years or older. Children under 16 years were encouraged to take part in the study and assent was taken based on their understanding.

Twenty-four participants were recruited into the study—eight from each age group 0–5, 6–10 and 11–18 years to provide a breadth of experience across the full childhood age range. The study was not offered to non-English speakers due to the short time opportunity to arrange an interpreter.

### Data collection

**Fig. 1:**
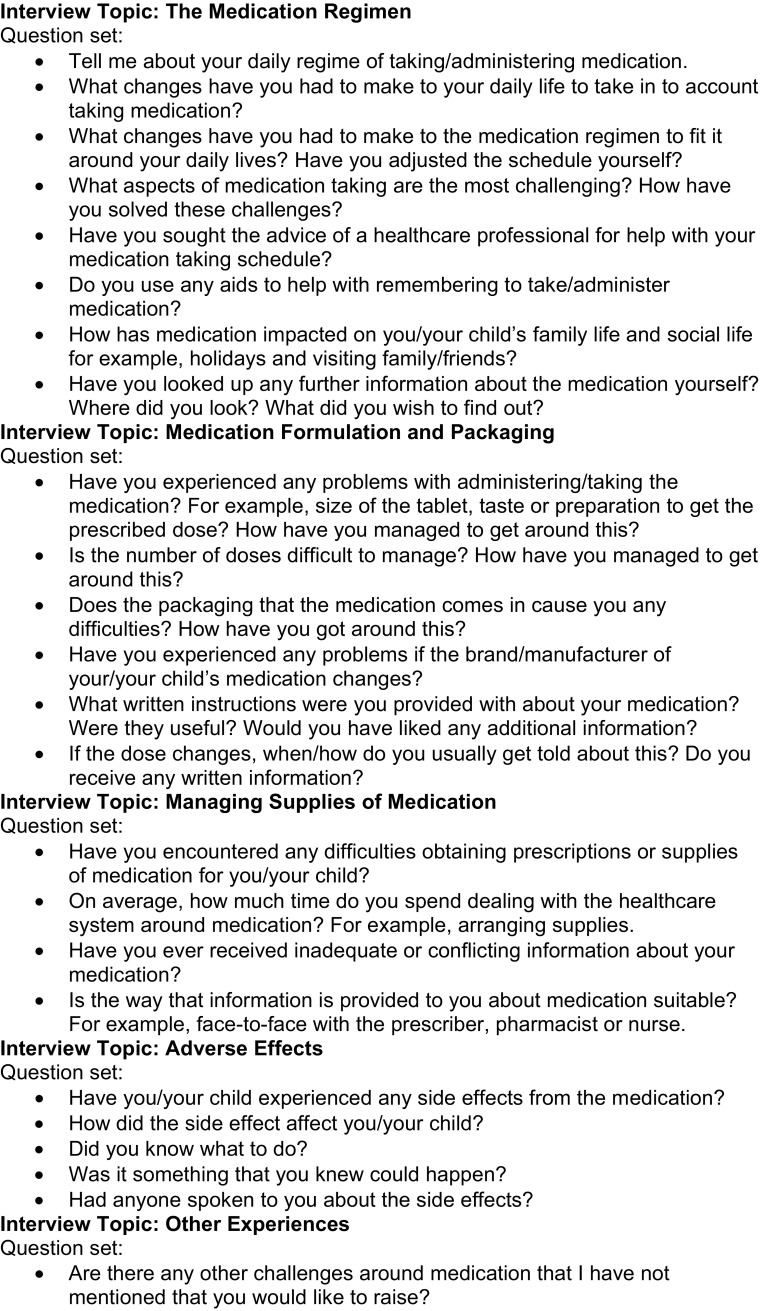
Interview questions

Face-to-face semi-structured interviews, with pre-piloted questions, were recorded and transcribed verbatim. The interviews took place during the patient’s in-patient stay and were undertaken by JA, a pharmacist not involved in the care of the study patients. The questions covered in the interviews were identified through a literature review (Fig. 1). Demographic/background information recorded included the patient’s age and usual medication.

### Data analysis

The interview transcripts were analysed using NVivo version 11. Thematic analysis was undertaken by JA using the 6 phases described by Braun and Clarke [16]. The themes identified were independently reviewed by KAW and DRPT.

## Results

Twenty-three parents and one 16-year-old patient consented. Assent was taken from 5 patients who contributed to the interviews with their parents. Two were aged 11 years, two 14 years and one 15 years.

In total 166 prescribed medications were taken by patients at home (Table 1). The number of medications prescribed for each patient ranged from 3 to 15 (mean 7, mode 5).

**Table 1:** Type of prescribed medication

Medication	Number prescribed
Vitamin and mineral supplementation	18
Antiepileptic	17
Treatment of gastro-oesophageal reflux disease	12
Inhaled bronchodilator	11
Treatment of constipation	11
Prophylactic antibiotics	9
Analgesia	8
Inhaled corticosteroid	6
Oral corticosteroid	6
Antiemetic	5
Nebulised sodium chloride	5
Oral antihistamine	4
Emollient	4
Pancreatin	4
Insulin	3
Nasal corticosteroid	3
Nebulised antibiotic	3
Nebulised DNase	3
Oral bronchodilator	2
Leukotriene antagonist	2
Other medications	30

Participants described many experiences of how taking medication impacted on their lives. These have been summarised into common themes. Participants identified additional experiences that were not part of the original interview framework. These included: the rigidity that parents demonstrated around dose times, managing dose changes in school, the internet as an information resource and for liaising with other parents and the influence of medication labelling.

### The timing of doses

Participants experienced challenges around the timing/frequency of doses. A four-times-daily regimen was the most difficult to adhere to due to the time available within daily activities. Participants described extending the duration of their day, arranging doses around meals/other medication and maintaining a precise time gap between doses.

We have to keep the gaps in-between equal, night-time especially because she has to have one at midnight, one at 2am then she’s due one at 6am. I have to stay up late until 2 o’clock and then I sleep after I’ve given her medicine. [Father of Patient 20 prescribed oral omeprazole, erythromycin, dexamethasone, glycopyrronium and co-trimoxazole.]

To make the medication regimen fit around daily life participants adjusted the timing of medication or daily activities. Establishing a routine was identified as important. Few participants sought advice about their medication schedule from a healthcare professional. Others sought advice on changing the timing of medication. This included adjusting the times away from the hospital administration times. Two participants had changed the regimen themselves.

### Medication at school

Participants described their experiences of medication at school. Whilst some had positive experiences others avoided the need to administer at school. Difficulties included educating teachers, administration in front of peers, transporting medication, limitations on frequency of administration and arranging additional medication for storage at school. School staff were unable to administer updated doses of medication following a verbal instruction of a dose change when medication was labelled with the previous dose.

If she’s gone to an out-patient appointment and her doses have changed she’ll have an old packet that hasn’t been labelled properly. Then I’m saying to [the school] the doses have changed. The school say ‘well we can’t give it because the dose that we’ve got is incorrect’. Then I’m waiting a week for the prescription to come or potentially two weeks for a letter from the hospital to get to the GP and then the GP to write out a new dose of medication. Quite often I’ll have to keep her off school because they can’t give the new dose. [Mother of Patient 21 prescribed oral desmopressin, levothyroxine, hydrocortisone and subcutaneous somatropin.]

### Medication adherence

Many participants cited remembering to give their child’s medication as their biggest challenge. Strategies employed to reduce the risk of unintended non-adherence included a mobile phone alarm, placing medication where it was visible, home-made chart/administration record and verbal reminders. A number of participants had purchased medication compliance aids. A second checking process had been adopted by one participant to reduce the risk of error.

I’ve had to put a list, like a checklist, on my fridge to make sure that I know I gave it him as well. I didn’t before and I used to feel like I was forgetting so I wrote it down so I know I gave it him. [Mother of Patient 14 prescribed oral multivitamins, vitamin E, ranitidine, pancreatin, nebulised sodium chloride, salbutamol and colistimethate.]

My husband and I always check them together to make it easier. In the past my mum did because she was a nurse and she taught me to double check which is brilliant because there have been times when I’ve been tired… [Mother of Patient 9 prescribed via gastrostomy senna, Movicol^®^, paracetamol, carbamazepine, levetiracetam, omeprazole, buccal midazolam, inhaled oxygen, rectal phosphate and sodium citrate.]

### Medication information

Participants found the information provided with their medication useful. Some described their experiences of receiving information about dose changes in clinic. This was provided verbally, with insufficient time for participants to write down, or with a hand-written note that was difficult to read.

That’s how we had to learn how to increase the dose. It was just a little scribble on a piece of paper from the consultant at first and the actual letter comes about three weeks later. I hope that when it finally comes through I’ve read this squiggle correctly and remembered what he said in clinic. [Mother of Patient 24 prescribed oral sodium valproate, carbamazepine and inhaled salbutamol.]

Few participants felt that they had received inadequate or conflicting information. Others believed that they were not told enough about access to medication outside of the hospital, how to use their medication, adverse effects or the type of medication prescribed.

We have some people telling us it’s really bad for him to be on [steroids]. When he’s older he’s going to suffer with his bones. When we went to the ‘out-of-hours’ at [the local hospital] it was one of the doctors there. So, we listen to him and then we’re told we need [the steroids by the respiratory team] so I’m like what do I do? [Mother of Patient 17 prescribed oral theophylline and montelukast. Inhaled salbutamol and Seretide^®^. Intranasal fluticasone.]

Participants commonly researched further information about their medication using the internet. This was for general interest, assurance, alternative treatment options, how to use their medication and information on adverse effects. Other participants avoided using the internet through fear of finding out something of concern.

At the end of the day we are responsible for [Patient 1]. I have researched them, I don’t understand half of it, but I’ve got an understanding to maybe ask the right questions and just check because we are responsible for him and we’ve not had anyone who’s on regular medicines in the family. [Mother of Patient 1 prescribed oral Movicol^®^, cetirizine, theophylline, hydrocortisone. Inhaled salbutamol and Seretide^®^. Intranasal fluticasone.]

Participants recounted experiences of using on-line support groups. Whilst helpful for some they created uncertainty for others through reading other patients’ experiences and advice from ‘expert parents’. One parent utilised a Facebook page for epilepsy and found that the reassurance provided reduced the need to contact the medical team.

I’ve joined a parenting group and I thought it would be nice to talk to other parents in the same position. They were saying things like if you give too much Creon then it will do this, you need to provide this sort of thing. I ignored it in the end and thought it’s probably best not to listen to you. Listen to the professionals. [Mother of Patient 14 prescribed oral multivitamins, vitamin E, ranitidine and pancreatin, nebulised sodium chloride, salbutamol and colistimethate.]

Different heart mum’s groups. They’ll say they were on captopril but now they’re on something and I’m like, well, what’s that then? Is it like captopril? Why is your daughter now taken off captopril and put on to this one and I’m thinking can’t [Patient 15] be taken off captopril and put on this one? [Mother of Patient 15 prescribed oral captopril and inhaled salbutamol (previously digoxin).]

I joined a parents’ for epilepsy Facebook. Sometimes you just think the doctor only has so much time with you and they have so much information that they can give you. It can be quite lonely out there when you don’t know what you’re doing. Reading about her hair falling out and the other mums and dads are saying it’s fine…it will grow back it’s not forever, she’s not going to end up completely bald. It can be reassuring. [Mother of Patient 24 prescribed oral sodium valproate, carbamazepine and inhaled salbutamol.]

### Medication formulation and packaging

Child resistance due to taste, colour, tablet size, refusal and disliking inhaler devices were cited. The ease of tablets compared with liquid formulations was mentioned.

It takes a lot more time to deal with liquids because you have to keep drawing them up. If I’m late for school, I can just grab a tablet and quickly take it. But when it comes to liquid I had to stay over a bit longer and draw it up. It’s more convenient with it being tablets. [Patient 2 prescribed oral phenoxymethylpenicillin, folic acid, paracetamol, ibuprofen and morphine sulphate.]

Parents used a variety of methods to aid their child’s medication taking. These included: distraction, tasting medication to empathise with their child, taste masking and changing the formulation.

Some participants had experienced difficulties with medication packaging and expressed concern about waste when receiving large bottles. Labelling caused some anxiety. A ‘cytotoxic’ label caused one participant to decide against taking their medication. An ‘unlicensed medication’ label on a bottle of phenobarbital caused concern.

I think the other thing was his phenobarbital coming with a great big label on saying ‘unlicensed medicine’. My mum saw it and she was like ‘oh my gosh! what are they doing?’. [Mother of Patient 11 prescribed oral phenytoin, vigabatrin, levetiracetam and ranitidine (previously phenobarbital).]

Few participants described challenges if the manufacturer of their medication changed. Others described uncertainty about whether they were receiving the correct medication, difficulty remembering the name, intolerance of alternative brands and the inconvenience of requiring refrigerated storage depending on the brand dispensed.

We have to try and keep to the same brand but we’ve found a lot of community pharmacists give us a different brand. Now the GP puts it on the prescription. Epilim liquid and syrup get interchanged. The syrup isn’t good for her teeth, it’s quite sugary and quite thick to [administer] to her. We do find one week we’ll get liquid and another we’ll get syrup. [Mother of Patient 24 prescribed oral sodium valproate, carbamazepine and inhaled salbutamol.]

### Travelling with medication

Administering medication was considered awkward in the presence of other people.

We’re in a café and we’re drawing up meds and everyone’s looking at you thinking ‘what are they doing!’. Especially when you’re out and about that’s the worst. [Mother of Patient 15 prescribed oral captopril and inhaled salbutamol (previously digoxin).]

Transportation was described as a problem for daily travel and holidaying. Particular problems were with refrigerated medication and large bottles. Some participants had purchased oral syringes with caps to carry doses. One participant risked the period of time that their refrigerated medication was transported at room temperature. Other participants used medication compliance aids for holidays and described using ice blocks to keep medication cool. Some participants avoided going on holiday due to the perceived difficulties over transporting and accessing medication.

Holidays is a hard one. When we got there we had a cold bag with ice packs in it and obviously the ice packs were melting and we had to stop and get ice from different shops. We had to stop at three different stops to get ice to cool his medicines down which was really hard. It was so hot the ice was melting and then when I got there the labels had come off! [Mother of Patient 18 prescribed oral sirolimus, mycophenolate, sodium bicarbonate, d-mannose and sodium feredetate.]

### Managing supplies of medication

Participants who received their medication through the hospital described the ease of receiving a prescription in an out-patient clinic and their medication from the hospital pharmacy. Those receiving medication through their GP highlighted community pharmacy prescription collection services and on-line ordering as useful. However, a number of participants described some difficulties obtaining medication in primary care. These were: the GP declining to prescribe, unavailability in community pharmacy, difficulties with the repeat prescription process and delayed communication between hospital and GP. Participants described the advanced planning that they undertook to maintain medication supplies.

Initially yes, it was a very big problem. Trying to get the GP to prescribe something that’s not listed in his bog standard BNF was a big issue. He refused to prescribe anything so now I literally don’t go to the GP. [Mother of Patient 18 prescribed oral sirolimus, mycophenolate, sodium bicarbonate, d-mannose and sodium feredetate.]

There’s certain meds the GP won’t prescribe. They’re like, ‘well, hang on they shouldn’t be on that med anyway’. That’s the way they see it. Even the digoxin, when we brought the forms to the GP after he got discharged he was looking at it and like ‘Really! Is he on that!? Are you sure!?’. [Mother of Patient 15 prescribed oral captopril and inhaled salbutamol (previously digoxin).]

The time taken to arrange supplies of medication focussed around two themes - ordering frequency and the time it took for the prescription and supply. In particular, having to frequently arrange supplies of medication due to a lack of synchronisation. This required ordering at least one medication weekly.

The phenobarbital in particular. We were told that we could order it and obtain it within 48 h. But subsequently actually we need 10 days. We’ve never run out but there was once in particular it was really challenging. [Mother of Patient 11 prescribed oral phenytoin, vigabatrin, levetiracetam and ranitidine (previously phenobarbital).]

### Adverse effects

Half of participants had experienced adverse effects ranging from diarrhoea to thrombocytopenia. Most had been informed by the healthcare team, other parents or through self-research. Most participants sought advice from nurses within their specialty. Mild side effects were managed by participants.

## Discussion

This study has identified many challenges that children, young people and their parents experience when a child or young person is taking regular prescribed medication. Similar experiences to those in the published literature were described including adherence, regimen inflexibility, impact on social activities, travelling with medication, administration at school and arranging repeat supplies. In addition, this study has identified how parents interpret dosing instructions, challenges around implementing dose changes in school and concern about medication waste.

The timing of doses and their impact on daily life was notable. The difficulty of the regimen has been shown to affect adherence in paediatrics improving once a routine is established [2]. In this current study participants had similar experiences but required support with the timing of administration, especially limiting this to waking hours. There are opportunities for this during the prescribing consultation, dispensing and medication review.

Challenges were identified with medication taken at school reflecting those previously described including access to medication and not wanting to take in front of peers [6, 17, 18]. Despite there being national guidance on medication in schools in the UK [19] and USA [20] poor experiences remain. A survey in Finland found inconsistencies in local school policies on medication [21]. This current study additionally identified difficulties around implementing dose changes. Information on changes to medication may be enhanced through the electronic transfer of clinic letters to GPs [22] and through direct electronic referral from hospital to community pharmacies [23]. Further work is required to support patients taking medication in school through better collaboration with healthcare professionals.

The most challenging aspect about having a child on medication was remembering to administer. The consequences of poor adherence are well established [1, 2]. A number of strategies were employed to aid adherence including compliance aids. The evidence base for medication compliance aids is limited and indicates a lack of patient benefit [24]. However, participants highlighted the additional benefit of compliance aids when transporting medication. This study highlights the importance of individualising patient care including considering the daily routine of each family.

Participants described receiving insufficient information in clinic verbally and through hand-written notes. The quality of instructions provided about medication influences adherence [1]. Healthcare practitioners may also influence adherence through patient engagement with conversations about medication [25]. Patients and parents require clear documentation of medication regimens.

Most participants looked up further information about their medication using the internet in accordance with published studies [26, 27]. Consultations with healthcare professionals are constrained by time [26]. A consequence of this is the desire to seek further information as explained by participants in this current study. However, poor interpretation of information about medications could lead to poor compliance [28]. A quality assessment tool may help children and parents to assess online information [28]. There is an opportunity at the points of prescribing and dispensing to ‘sign-post’ people to quality assured internet sites.

Some participants accessed on-line parent support groups which is observed in parents/patients with long-term conditions [26–28]. Some found these groups informative whereas others found they raised more questions and disliked the ‘expert parent’ approach. Further research has been suggested around how pharmacists may support patients using the internet for medication information [28].

The absence of child friendly formulations was problematic. To optimise the use of currently available formulations, training in swallowing medication could be provided by healthcare professionals which has previously proved successful [29].

Participants expressed concern about wasted medication. In the UK approximately £300 million of NHS prescribed medication is wasted annually [30]. A recent study identified that more than 33% of medication returned to Dutch community pharmacies was preventable [31]. Globally, the total amount of medication consumed will increase by about 3% through 2021 with spend approaching $1.5 trillion [32]. Therefore, initiatives that have been described to reduce waste [30] will be of increasing importance. This study confirms that medication waste is evident in paediatrics with parents expressing concern. There are opportunities for pharmacists to reduce waste through medication review.

Travelling with medication and taking medication outside of the home proved challenging. Parents were making decisions around the stability of medication out of the fridge and usual packaging. This current study identified that more support and advice is required for parents/patients travelling with medication.

Challenges were described arranging supplies of medication in primary care as previously described [33]. These remain problematic for parents and patients who may not be informed of these potential problems. Contact between the hospital and the patient’s GP to agree the supply route should take place at the earliest opportunity. Better integration of pharmacists and GP working can optimise medication supply including synchronising repeat medications [34]. Timely transfer of information is recommended as a standard for good medicines optimisation [35].

Participants reported adverse effects from their medication. Treatment side effects have been shown to be a factor in non-adherence in paediatrics [2]. Parents and patients should be informed about potential adverse effects, their management and how to seek advice. There remains further opportunity to understand how patients and parents would like to be informed about adverse effects.

The strength of this study is the detailed insight into how medication taking in children impacts on daily life from the perspective of the parent and/or the patient. The results from the study can be incorporated in prescribing and dispensing consultations to further optimise medication use. These findings may also be incorporated in a formal paediatric medication review with individual patients/parents.

Study limitations include the possibility of participants providing answers that they perceived to be acceptable. Consistency of the interview process was maintained with one researcher undertaking all interviews. The interviews took place whilst the patient was an in-patient which may have influenced how participants prioritised their experiences. Undertaking the research at a single UK institution may limit the generalisability of the results. Whilst healthcare systems differ between countries, many of the experiences investigated are likely to be similar.

## Conclusion

Parents and patients experience many challenges with their medication. This study has identified the following opportunities for healthcare professionals to contribute towards the optimal use of medication in paediatric patients:

- Engagement with patients and parents regarding medication choice/regimen to ensure treatment is achievable within their daily lives.
- Better collaboration with schools regarding patients’ medication especially when changes are made to treatment.
- Provision of clear instructions regarding changes that patients/parents are expected to make to current treatment.
- Sign-posting to quality assured internet sites about medication.
- Provide support to children to swallow solid dose forms.
- Ensure medication quantity is optimised to reduce waste.
- Early collaboration between hospital and primary care health providers to agree medication supply.

Minimally disruptive medication tailored to the realities patients’ daily lives could greatly improve quality of life [15]. This current study has identified how medication taking affects daily life when children and young people take regular medication.
